# Single-cell RNA sequencing reveals small extracellular vesicles derived from malignant cells that contribute to angiogenesis in human breast cancers

**DOI:** 10.1186/s12967-023-04438-3

**Published:** 2023-08-25

**Authors:** Youxue Zhang, Fang Zhen, Yue Sun, Bing Han, Hongyi Wang, Yuhang Zhang, Huaixi Zhang, Jing Hu

**Affiliations:** 1 Department of Breast Medical Oncology, Harbin Medical University Cancer Hospital, Harbin, Heilongjiang China; 2 Department of Breast Surgery, Harbin Medical University Cancer Hospital, Harbin, Heilongjiang China

**Keywords:** Breast cancer, Angiogenesis, Single-cell RNA sequencing, Small extracellular vesicles, Endothelial cells

## Abstract

**Background:**

Breast cancer is the most common cancer affecting women across the world. Tumor endothelial cells (TECs) and malignant cells are the major constituents of the tumor microenvironment (TME), but their origin and role in shaping disease initiation, progression, and treatment responses remain unclear due to significant heterogeneity.

**Methods:**

Tissue samples were collected from eight patients presenting with breast cancer. Single-cell RNA sequencing (scRNA-seq) analysis was employed to investigate the presence of distinct cell subsets in the tumor microenvironment. InferCNV was used to identify cancer cells. Pseudotime trajectory analysis revealed the dynamic process of breast cancer angiogenesis. We validated the function of small extracellular vesicles (sEVs)-derived protein phosphatase 1 regulatory inhibitor subunit 1B (PPP1R1B) in vitro experiments.

**Results:**

We performed single-cell transcriptomics analysis of the factors associated with breast cancer angiogenesis and identified twelve subclusters of endothelial cells involved in the tumor microenvironment. We also identified the role of TECs in tumor angiogenesis and confirmed their participation in different stages of angiogenesis, including communication with other cell types via sEVs. Overall, the research uncovered the TECs heterogeneity and the expression levels of genes at different stages of tumor angiogenesis.

**Conclusions:**

This study showed sEVs derived from breast cancer malignant cells promote blood vessel formation by activating endothelial cells through the transfer of PPP1R1B. This provides a new direction for the development of anti-angiogenic therapies for human breast cancer.

**Supplementary Information:**

The online version contains supplementary material available at 10.1186/s12967-023-04438-3.

## Introduction

Angiogenesis is the biological process which new blood vessels are formed, a complex and highly ordered mechanism involving a variety of cellular processes, including the degradation of the vascular basement membrane, endothelial cell activation, proliferation, and migration [1]. Angiogenesis is essential to carcinogenic processes, including solid tumor formation, growth, invasion and metastasis. The “angiogenic switch” occurs when dormant tumor cells activate angiogenesis by secreting factors that induce endothelial cells to germinate and chemotaxis into the tumor mass, which activates the expression of several genes responsible for angiogenesis [2, 3].

Breast cancer is the most diagnosed form of cancer in women worldwide [4]. Current treatments for breast cancer include surgery, chemotherapy, and endocrine, targeted and radiation therapies, which encompass anti-angiogenic therapy [5, 6]. These treatment methods improved the survival and prognosis of patients with breast cancer, but the positive outcomes remain limited [7]. At present, anti-angiogenic drugs are used as first-line treatment of advanced breast cancer, including Bevacizumab, Apatinib, Anlotinib, Endostar, Ramucirumab, Sunitinib, Sorafenib. Clinically, higher levels of angiogenesis lead to worse prognosis, and several patients may possess sensitivity to anti-angiogenic drugs [8–10]. Accordingly, it is necessary to explore tumor heterogeneity and discover genes that drive angiogenesis.

Extracellular vesicles, known as exosomes, are nanoscale bilayer extracellular vesicles with an average diameter of 40–160 nm that can be released by most cells in the tumor microenvironment [11]. Extracellular vesicles function as information and communication vessels between various cell types and are involved in the regulation of the tumor microenvironment, such as metastasis, immune escape, drug resistance and angiogenesis [12, 13].

We used single-cell transcriptome profiling to characterize cancer cells and the heterogeneity of TECs and unraveled an atlas of angiogenesis in human breast cancer. Our work highlights the role of TECs subsets in angiogenesis and identifies possible therapeutic targets for breast cancer treatment. In addition, we phenotypically classified malignant cells and endothelial cells in the tumor microenvironment. Through the analysis of the heterogeneity of tumor cells and TECs explored the potential biological functions of tumor cells and in the progression of tumor angiogenesis and found that tumor cell-derived extracellular vesicles may promote tumor angiogenesis by transferring some cargos to endothelial cells by scRNA-seq. This study provides a valued resource for uncovering the intra-tumoral heterogeneity of breast cancer, revealing the developmental process of breast cancer angiogenesis, and laying the foundation for anti-angiogenic therapy for breast cancer.

## Materials and methods

### Tissue samples

A total of eight breast invasive ductal carcinoma samples were collected at the Harbin Medical University Cancer Hospital. Patient clinical information is available in Additional file 2: Table S1.

### Single cell collection

Samples from eight patients were analyzed from fresh surgical tissues. Tumors were dissociated using the Human Tumor Dissociation Kit (#130-095-929, Miltenyi Biotech, Germany) according to the manufacturer’s protocol [14]. The digested tissues were passed through 70 μm SmarterStrainers (#130-098-462, Miltenyi Biotech, Germany), and the suspended cells were centrifuged at 300 × g for 7 min. After eliminating dead cells, cell suspensions were directly used for single-cell RNA-seq, as described below.

### Single-cell library preparation and sequencing

Cellular suspensions were loaded onto a Chromium Single Cell Instrument (10X Genomics) to generate single-cell gel beads in emulsions. We then amplified cDNA by PCR. All samples were processed in parallel in the same thermal cycler. The cell suspensions were converted to barcoded RNA-seq libraries using the Chromium Single Cell 5'v3 reagent kit (10X Genomics, USA) as per the manufacturer’s protocol, aiming for 10,000 cells per library. All samples were sequenced on an Illumina HiSeq2000 sequencing platform.

### ScRNA-seq data processing and quality control

The Bcl2fastq (v2.17.1.14) software was used to identify raw sequence data based on images. The sequencing results in the original image data were stored in the FASTQ file format. FastQC (version 0.10.1) was used for quality analysis of the sequence data. Cutadapt (version 1.9.1) was used to remove linkers and low-quality sequences from the raw data and analyze the resulting information. The scRNA-seq raw data was aligned to the Genome Reference Consortium Human Construction 37 (GRCh37) using the BWA (version 0.7.12) software with default parameters.

We used the Hisat2 (version 2.0.1) software with default parameters to analyze Cutadapt short-read filtered data. The corresponding results were stored in the SAM file format and converted to BAM using SAMtools. Expression profiles were obtained using FeatureCount and normalized with the R package 'limma'.

Single cells were filtered for downstream analysis based on SeuratQC with the following criteria: unique molecular identifier (UMI) count between 3,000 and 40,000, and the proportion of mitochondrial UMI < 10%. Gene expression was normalized using SeuratNorm. A total of 48,644 single cells passed the QC criteria and were used for further analysis.

### Single-cell landscapes constructions and annotation of cell clusters

The standardized processing of single-cell data was achieved with Seurat. We employed the standardized function sctransform in the Seurat package in R. Sctransform modeled single-cell UMI expression data using regularized negative binomial regression to eliminate variation due to sequencing depth, preserving true biological heterogeneity [15].

We constructed the cell clusters and single-cell atlas using Seurat with default parameters in R. We used uniform manifold approximation and projection (UMAP) for dimension reduction and visualization. The FindAllMarkers function from the Seurat R package was used to identify marker genes highly expressed in each cell cluster. Cell clusters were then classified according to the expression of canonical cell type marker genes from the Cell Markers database.

### CNV estimation

We identified copy number variations (CNVs) from transcriptomic profiles on scRNA-seq data using the inferCNV package (version 1.1.3) [16]. The inferCNV package compared gene expression in each tumor cell to reference gene expression in other cells. We visualized large-scale copy number variations in scRNA-seq expression data using inferCNV package.

### Pseudotime trajectory analysis

Pseudotime analysis was performed with R package Monocle3 (version 1.0.0) to investigate the relationships between cell types and different clusters [17]. The goal was to characterize functional changes in malignant cells and determine potential lineage differentiation. Further detection with Monocle3 pseudotime function revealed the key role of some genes in the progression of angiogenesis.

### SCENIC analysis and transcription factor-target gene network analysis

Single-cell regulatory network inference and clustering (SCENIC) (version 1.1.0.1) was used to analyze gene regulatory networks and identify cellular states based on single-cell expression profiles, providing significant biological insights into the underlying mechanisms associated with cellular heterogeneity [18]. To identify internal transcriptional regulatory drivers of angiogenesis in breast cancer, we used the Python module tool pySCENIC to analyze and reconstruct gene regulatory networks with transcription factors (TFs) as the core.

### GO and KEGG analysis

Gene Ontology (GO) and Kyoto Encyclopedia of Genes and Genomes (KEGG) enrichment analyses were applied to determine the underlying function and molecular pathways occurring in each cell subpopulation using the R package clusterProfiler (*P* < 0.05).

### Cell culture and transfection

T47D and MDA-MB-231 cell lines were maintained in our laboratories (Cancer Research Institute, Harbin Medical University). The T47D cell line was cultured with DMEM containing 10% FBS in 5% CO_2_ at 37 °C. The MDA-MB-231 cell line was cultured in L15 supplemented with 10% FBS at 37 °C. Cells were authenticated by short tandem repeat (STR) sequence analysis. All experiments were performed with Mycoplasma-free cells. 2 × 10^5^ cells were inoculated in 6-well plates. Knockdown and overexpressed lentivirus were transfected into the cells containing 5 µg/mL polybrene. Stable cells were selected with 2 µg/mL puromycin.

### Reverse transcription and quantitative real-time PCR

In this experiment, total RNA was extracted from the cultured cells using TRIzol reagent (Roche). Then, 1000 ng of RNA from each sample was reverse-transcribed into cDNA using the Transcriptor First Strand cDNA Synthesis Kit (Roche). Quantitative analysis of the target genes was performed using SYBR^®^ Green Real-time PCR Master Mix (Roche). Each sample was tested in triplicate, and the changes in gene expression were calculated based on the threshold cycle (Ct) values between the target genes and GAPDH, following the manufacturer's user manual.

### Western blot analysis

Western blot analysis was performed previously as described [19]. We used the following primary antibodies: CD63 (#ab134045, abcam); CD9 (#13403, Cell Signaling Technology); TSG101 (#ab83, abcam); calnexin (#2679, Cell Signaling Technology); PPP1R1B (#ab40801, abcam).

### Tube formation

300 µl of Matrigel (Corning, USA) was spread across a 24-well plate on ice. The plate was incubated at 37 °C for 30 min. 300 µl of HUVECs suspension (1.2 × 10^5^) incubated with different sEVs was added to each well. After 6 h of incubation, tube formation was imaged on a microscope. The total branching lenght was analyzed by Image J. Results from three independent experiments are represented as the mean ± SD.

### Wound healing and transwell migration and invasion assays

The cells were grown in 6-well plates until they formed a complete layer covering the bottom of each well. Microtubule tips were used to scrape vertically across the cell layer to create a wound. The culture medium was then replaced with a serum-free medium. After 24 h, the rate of wound healing was observed and photographed. In the transwell assay, 5 × 10^4^ cells were suspended in 200 µl of serum-free culture medium and added to the upper chamber of the transwell. After incubating for 24–48 h (24 h for migration assay), the cells that had migrated to the bottom surface of the membrane were fixed and stained with crystal violet. Five randomly selected fields were photographed for statistical analysis per well.

### Isolation and identification of extracellular vesicles

The FBS was depleted of EVs by differential ultracentrifugation at 100,000 × g for 18 h at 4 °C [20]. Extracellular vesicles were separated from the EV-depleted medium by differential ultracentrifugation [21]. First, the medium was centrifuged at 300 × g for 10 min at 4 °C. After this, the supernatant was centrifuged at 2000 × g for 10 min at 4 °C and 10,000 × g for 30 min at 4 °C. The resulting supernatant was then ultracentrifuged at 100,000 × g at 4 °C for 70 min, washed with commercial PBS and centrifuged at 100,000 × g for another 70 min. Extracellular vesicles were confirmed by transmission electron microscope (TEM) and NanoSight Tracking analysis (NTA) before resuspension in PBS. The interaction of tumor-derived sEVs with endothelial cells was observed and labeled with PKH26 (Sigma).

### Immunofluorescence staining

Immunofluorescence staining was employed to investigate protein expression and examine the subcellular localization of SRGN, S100A9, PPP1R1B, CXCR4, and AGR3. Tumor tissues were deparaffinized and rehydrated, followed by antigen retrieval. After 30 min blocking in 5% bovine serum albumin (BSA) at 37 °C, tissues were incubated with the following primary antibodies: EPCAM (#BF0159, Affinity), CD31 (#BF0611, Affinity), SRGN (#BS-6789R, Bioss), S100A9 (#DF7596, Affinity), PPP1R1B (#T55374, Abmart), CXCR4 (#AF5279, Affinity), and AGR3 (#11967-1-AP, Proteintech) at 4 °C overnight. The secondary antibodies were subsequently added for 1 h at room temperature followed by counterstaining with DAPI (#AR1176, BOSTER). The tissues were then observed and photographed under a fluorescence microscope (Zeiss, Germany), briefly, we stained the genes and endothelial and breast cancer cell markers to confirm the co-existence of these markers.

### Statistical analysis

Statistical analyses were performed using R (version 4.1). Data analysis was performed using GraphPad Prism 8. The data are presented as the mean ± standard deviation (SD) values. Student’s t-test and ANOVA were utilized to calculate the significance of differences between groups. The survival analysis was performed using the Kaplan–Meier analysis. All experiments were repeated at least three times. Statistical results with *P* < 0.05 indicated statistically significant.

## Results

### Single-cell atlas of angiogenesis in human breast cancer

**Fig. 1:**
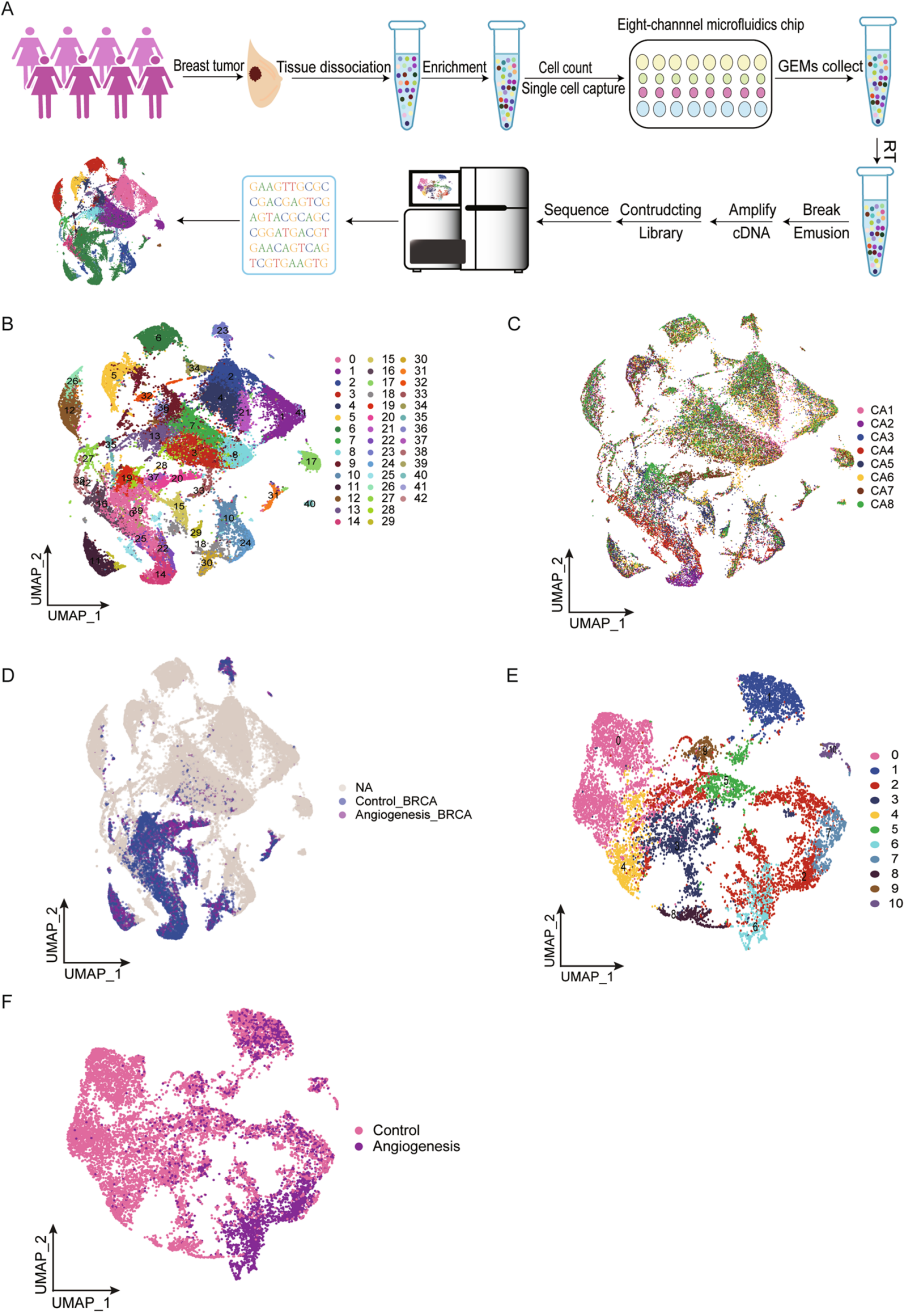
Single-cell transcriptomic profiles of breast cancers with and without angiogenesis. **A** Workflow illustrating sample preparation, sequencing and bioinformatic analysis. **B** UMAP plot colored by inferred cell clusters. **C** UMAP of 48,644 cells from eight breast cancer tissues. **D** The distribution of malignant cells in different tumors with and without angiogenesis. Malignant cells with or without angiogenesis were classified as angiogenesis and control groups, respectively. The rest of the cells were classified in the NA group. **E** Single-cell map showing subclusters of malignant cells. **F** Single-cell atlas showing tumor cell subsets of angiogenic and non-angiogenic breast cancer groups

To dynamically dissect the evolution and molecular signatures during clonal breast cancer evolution, we profiled the transcriptome of each cell population using single-cell RNA sequencing. We focused on exploring the process of angiogenesis in breast cancer by analyzing eight fresh human breast cancer tissue samples (CA1-8) using scRNA-seq. Vascular dysfunction acts as a key role in cancer metastasis [22]. CD31, also known as platelet endothelial adhesion molecule 1 (PECAM1), is commonly as a marker of endothelial cells to demonstrate the presence of endothelial tissue and evaluate tumor angiogenesis [23]. We thus examined the expression of CD31 in tumor tissue using immunohistochemical staining to estimate angiogenesis. We found that the immunohistochemical staining of CD31 was positive in two samples (CA6, CA7), but negative in the other six samples (Additional file 2: Table S1). Tumor tissues were digested to single-cell suspensions, sorted for viability, and profiled using the 10X Genomics protocol (Fig. 1A). After quality control, we obtained 48,644 high-quality single cells, which were annotated with canonical lineage markers [24]. We used UMAP visualization to initially divide these cells into 43 major clusters, and mapped single cells from eight patients (Fig. 1B, C). The distribution of each cell cluster varied greatly among patients with and without angiogenesis as measured by the Seurat package (Fig. 1D, Additional file 1: Figure S1). Single-cell map showed subclusters of malignant cells (Fig. 1E, F). In summary, our results revealed differences in cell types and tumor tissue composition among breast cancer patients. The heterogeneity of tumor tissues was consistent with previous reports [25, 26].

### Intrinsic malignant cell subclones underlying tumor subtypes

**Fig. 2:**
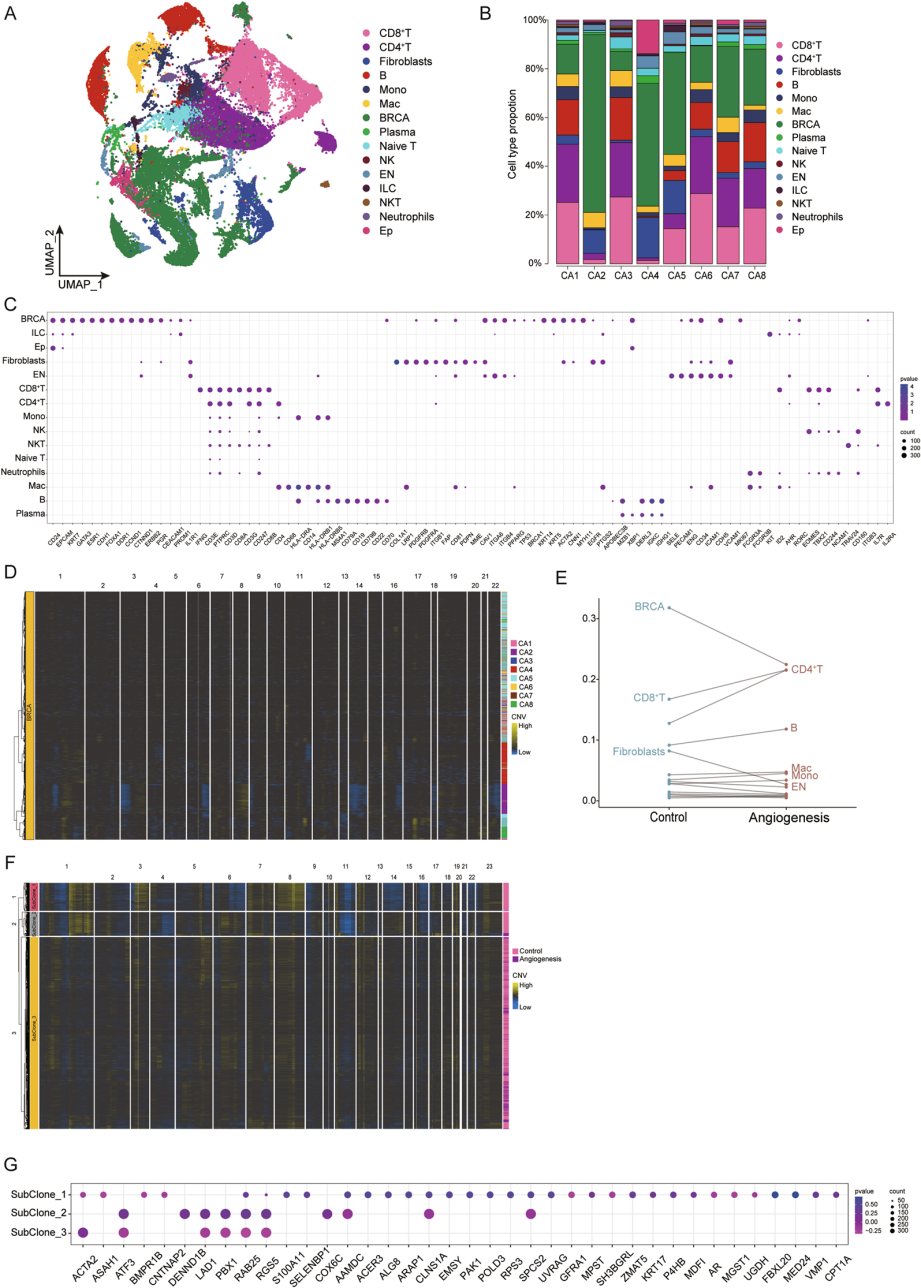
Subclones of malignant cells underlying tumor subtypes. **A** A UMAP view of 48,644 cells, color-coded by assigned cell type. **B** Proportion of predominant cell types in each patient. **C** Expression levels of specific marker genes in each cell subtype. **D** InferCNV heatmap for malignant cells showing significant copy number variations across tumors. **E** The distribution of each cell type among different tumor pathological subtypes. **F** InferCNV showing the subclones of malignant cells. **G** Bubble map depicting the expression levels of marker genes in CNV subclones

According to the expression of canonical lineage markers from the scRNA-seq profiles, these cells were grouped in fifteen cell clusters, which were termed CD8^+^ T cells, CD4^+^ T cells, fibroblasts, B cells, mononuclear leucocytes, macrophage, breast cancer cells, plasma cells, naive T cells, natural killer cells, endothelial cells, intrinsic lymphocytes, natural killer T cells, neutrophils and epithelial cells [27]. A total of 48, 644 single cells from tumor tissues were visualized using UMAP (Fig. 2A, Additional file 1: Figure S2). The internal cell composition of different patients was analyzed (Fig. 2B), and the cell components of tissue samples showed substantial heterogeneity. We used a bubble plot to reveal the top differentially expressed genes in each cluster (Fig. 2C).

ScRNA-seq became available to identify the presence of normal cells in the tumor microenvironment and characterize the expression of tumor cells in a variety of human cancers. We identified single-cell copy number variant profiles using InferCNV and found copy number differences among angiogenic and non-angiogenic tumor cells (Fig. 2D). We also identified all major cell types across tumors and found significant differences in cell type abundance between angiogenesis negative and angiogenesis positive tumors (Fig. 2E, Additional file 1: Figure S3). Our analysis further demonstrated the existence of different CNV subclones in malignant cells, implying that the structure of tumor angiogenesis may be determined by dynamic evolutionary processes (Fig. 2F). The differences in gene expression in malignant cells regulated by CNVs was shown as a bubble plot (Fig. 2G).

These results show that inferCNV can resolve clonal copy number substructures from scRNA-seq data, and identify subclonal differences in breast cancer genes and cancer phenotypes present within the tumor masses.

### Identification of malignant cell subgroups involved in angiogenesis

**Fig. 3:**
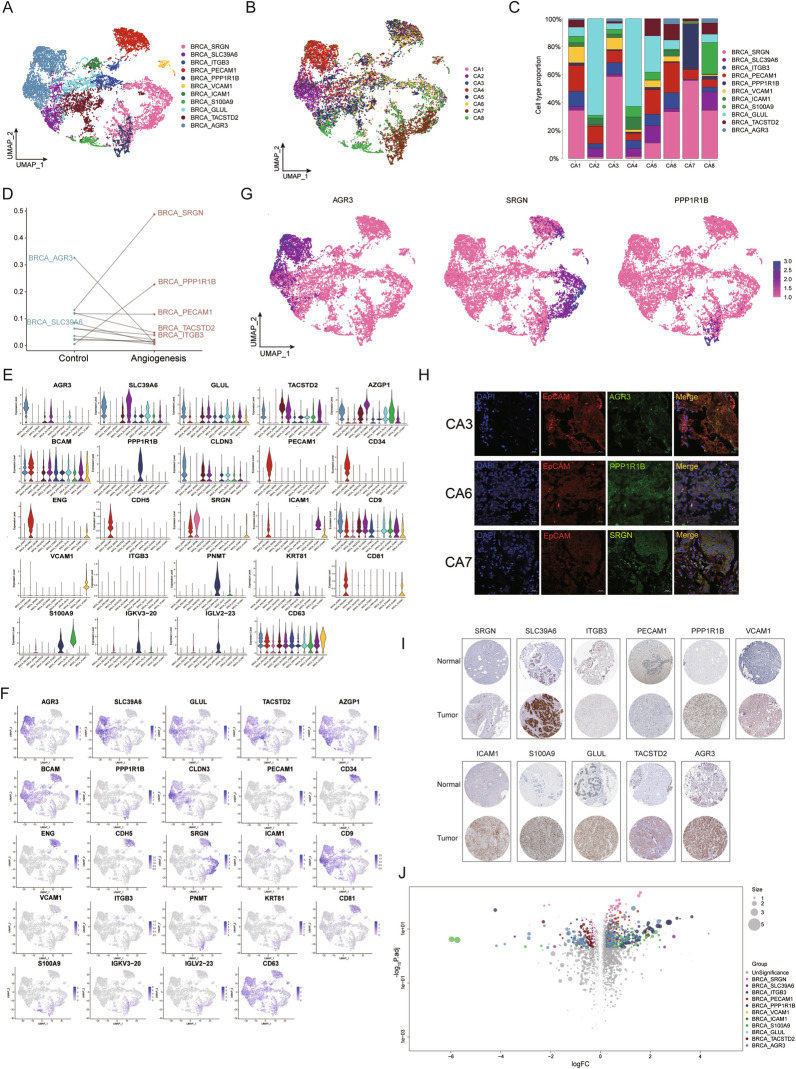
Differences between subsets of malignant cells with and without angiogenesis. **A** Single cell map showing eleven malignant cell subclusters. **B** UMAP plot of 14,151 cells from eight patient samples. **C** Tumor cell subclusters composition according to the patients. **D** Scatter plot showing the distribution of tumor cells in angiogenic and non-angiogenic breast cancer. **E**–**F** Violin plots and UMAP figures showing malignant cell-related markers and their distribution in malignant cell subgroups. **G** UMAP series of single-cell maps of BRCA_AGR3, BRCA_SRGN and BRCA_PPP1R1B. **H** Immunofluorescence imaging of AGR3, SRGN, PPP1R1B (green) and EpCAM (red) in three breast cancer tissue samples. The nuclei were counterstained with DAPI. Scale bar, 20 µm. **I** Representative images of the IHC of key genes between breast cancer and normal breast tissues available in the HPA database. **J** Volcano plot showing differences in gene expression genes in eleven tumor cell subclusters

We next evaluated whether the transcriptome of tumor cells may show indications of tumor angiogenesis. Accordingly, tumor cells were extracted for subpopulation analysis, and the identified malignant cells clustered into eleven subclones: BRCA_SRGN, BRCA_SLC39A6, BRCA_ITGB3, BRCA_PECAM1, BRCA_PPP1R1B, BRCA_VCAM1, BRCA_ICAM1, BRCA_S100A9, BRCA_GLUL, BRCA_TASTD2 and BRCA_AGR3 (Fig. 3A, Additional file 1: Figure S4). We performed basic Seurat processing analysis by combining gene-specific expression markers for angiogenic and non-angiogenic breast cancer and CNVs for malignant subclones as custom genes. Breast cancer cells from eight patients were visualized using UMAP (Fig. 3B), and the malignant cell subcluster composition of each tumor differed substantially (Fig. 3C). In addition, we observed that BRCA_AGR3 was highly expressed in the angiogenesis-negative group; BRCA_PPP1R1B was specifically highly expressed in the angiogenesis positive group; and BRCA_SRGN was highly expressed in both groups (Fig. 3D, Additional file 1: Figure S5). CD63, a transmembrane 4 superfamily protein, is a key factor regulating extracellular vesicle production and intracellular cargo sorting that is mostly used to identify exosomes [11]. We identified several malignant cell markers and used UMAP to infer the distribution of these subclusters in the malignant cell atlas (Fig. 3E, F). UMAP showed the subcluster distribution of BRCA_AGR3, BRCA_SRGN and BRCA_PPP1R1B (Fig. 3G), which have been associated with cancer cell progression and chemotherapy resistance [28–30]. Specifically, AGR3 promotes tamoxifen resistance in breast cancer [31], SRGN is a key molecule in mediating chemoresistance and stemness in breast cancer cells [30], and PPP1R1B is involved in resistance of breast cancer cells to trastuzumab [32].

Epithelial cell adhesion molecule (EpCAM, also known as CD326) is a single-channel type I plasma membrane glycoprotein expressed in a variety of tumor epithelial cells that is commonly used as primary tumor cell marker [33, 34]. We conducted immunofluorescence staining to confirm the distribution of malignant cell subclones in tissues (Fig. 3H), and obtained immunohistochemical images of these eleven markers in breast cancer tissues from the Human Protein Atlas database (HPA) (Fig. 3I). Our results showed that the marker genes of malignant cell subclusters had a higher expression in breast cancer than normal breast tissues, confirming the existence of malignant cells subgroups in the former.

We next analyzed gene expression changes among malignant cells subclusters, and found that gene expression in angiogenic breast cancer was significantly different from non-angiogenic breast cancer (Fig. 3J), indicating that the presence of specific genes involved in tumor angiogenesis and metastasis. Further studies on these specific angiogenic markers are necessary for the development of anti-angiogenic therapies for breast cancer.

In conclusion, it is obvious that the expression of gene signatures was significantly different between the angiogenesis-positive tumor and the angiogenesis-negative group. These results indicated that some specific genes were involved in angiogenesis in the process of tumor and metastasis. Further studies on these specific marker genes of angiogenesis are necessary for anti-angiogenic therapy of breast cancer.

### Clonal evolution of angiogenic breast cancer cells

Because tumor cells induce the formation of blood vessels in the absence of oxygen, we employed a cell evolution trajectory to study the relationship between angiogenesis and tumor cell differentiation.

**Fig. 4:**
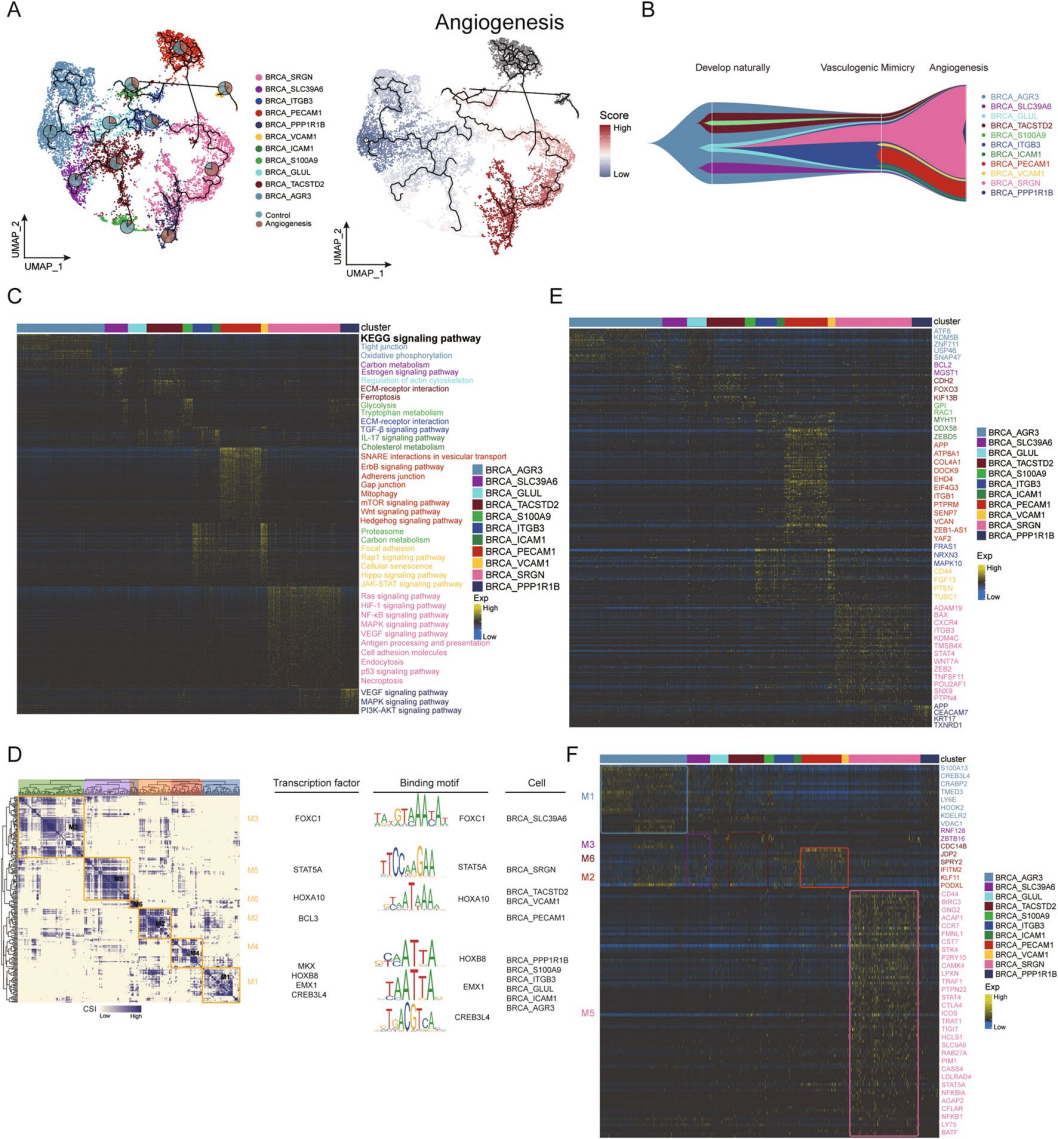
Taxonomy and developmental trajectory of malignant breast cancer cells. **A** BRCAs were ordered according to pseudotime trajectories and color-coded by cluster. Pie charts showing changes in malignant cell subclusters in the angiogenesis and non-angiogenesis groups. **B** Fish plot of the clonal lineages of major tumor subclones. **C** Heatmap-pathways showing genes regulated by malignant subclonal cells and signaling pathways. **D** Identification of regulon modules, along with representative transcription factors, corresponding binding motifs, and associated cell types. **E** InferCNV profiles of malignant cell subclusters. Cell-specific CNVs regulated the expression of genes involved in the evolution of angiogenic phenotypes in tumor cells. **F** Heatmap showing the transcriptional expression regulation of cell-specific TFs

This was achieved using pseudotime sequence trajectory (starting from the clusters with the highest proportion of non-angiogenic breast cancers), which was consistent with the trend of angiogenesis score. Pie charts characterized the proportion of angiogenesis negative and positive malignant cell clusters at different pseudotime values (Fig. 4A). The angiogenesis index score and pseudotime trajectory analysis revealed the angiogenic process in eleven BRCA clusters, with the identified structural changes in malignant cells through natural development, vasculogenic mimicry, and angiogenesis (Fig. 4B). Strikingly, BRCA_AGR3 was identified at an early stage of the angiogenesis. BRCA_PPP1R1B and BRCA_SRGN were in advanced stages, suggesting that they may be highly correlated with tumor angiogenic phenotype. Consistent with our previous finding, BRCA_AGR3 was highly expressed in non-angiogenic breast cancer samples, while BRCA_PPP1R1B and BRCA_SRGN were highly expressed in angiogenic breast cancer samples in Fig. 3D. Clonal phylogenetic analysis revealed dynamic characteristics and heterogeneity in cell populations, further confirming the evolutionary relationship. These BRCA clusters might drive tumor angiogenesis at different stages. The heatmap pathway displayed quasi-time-dependent genes associated with the evolution of regulating angiogenic phenotype in tumor cells and the relative pathway enrichment analysis (Fig. 4C). Moreover, BRCA_SRGN and BRCA_PPP1R1B were enriched in cell adhesion molecules, VEGF and MAPK signaling pathways, which further confirmed the central role of BRCA_SRGN and BRCA_PPP1R1B in tumor angiogenesis. Next, we used SCENIC to identify effectively six major co-expression modules between transcription factors and potential target genes. The gene regulatory network (GRN) plays an important role in the regulation of gene expression. For each module, we identified several representative TF regulons, corresponding binding motifs, and cell types. We highlighted six modules named M1-M6 and found that each module occupied a different domain. M1 contains regulators that were correlated with cell differentiation, such as CREB3L4, EMX1, MKX and HOXB8. M1 is related to BRCA_ITGB3, BRCA_PPP1R1B, BRCA_ICAM1, BRCA_S100A9, BRCA_GLUL, and BRCA_AGR3. M2 is connected with BRCA_PECAM1 and contains BCL3, which promotes cell proliferation and inhibits apoptosis [35, 36]. Regulons in M3, including FOXC1, is associated with BRCA_SLC39A6. M5 includes the regulator STAT5A, which is associated with BRCA_SRGN. M6 contains regulators HOXA10, and is associated with BRCA_TASTD2 and BRCA_VCAM1 (Fig. 4D). InferCNV was applied to analyze the copy number variations in cancer cell subclusters. To demonstrate that cell-specific CNV regulates the expression of genes related to the evolution of angiogenic phenotype in tumor cells, FindMarkers was used to identify CNV marker genes of malignant cell clusters and cell-specific TF regulating the transcriptional expression of genes related to the evolution of angiogenic phenotypes in tumor cells were presented, respectively (Fig. 4E, F).

Therefore, we inferred the origin and trajectory of clonal evolution through which breast cancer cells acquire an angiogenic phenotype, and identified the genes involved in the evolution processing and their global network of copy, transcription, and post-transcriptional regulation.

### Identification of diverse subgroups of endothelial cells associated with breast cancer angiogenesis

**Fig. 5:**
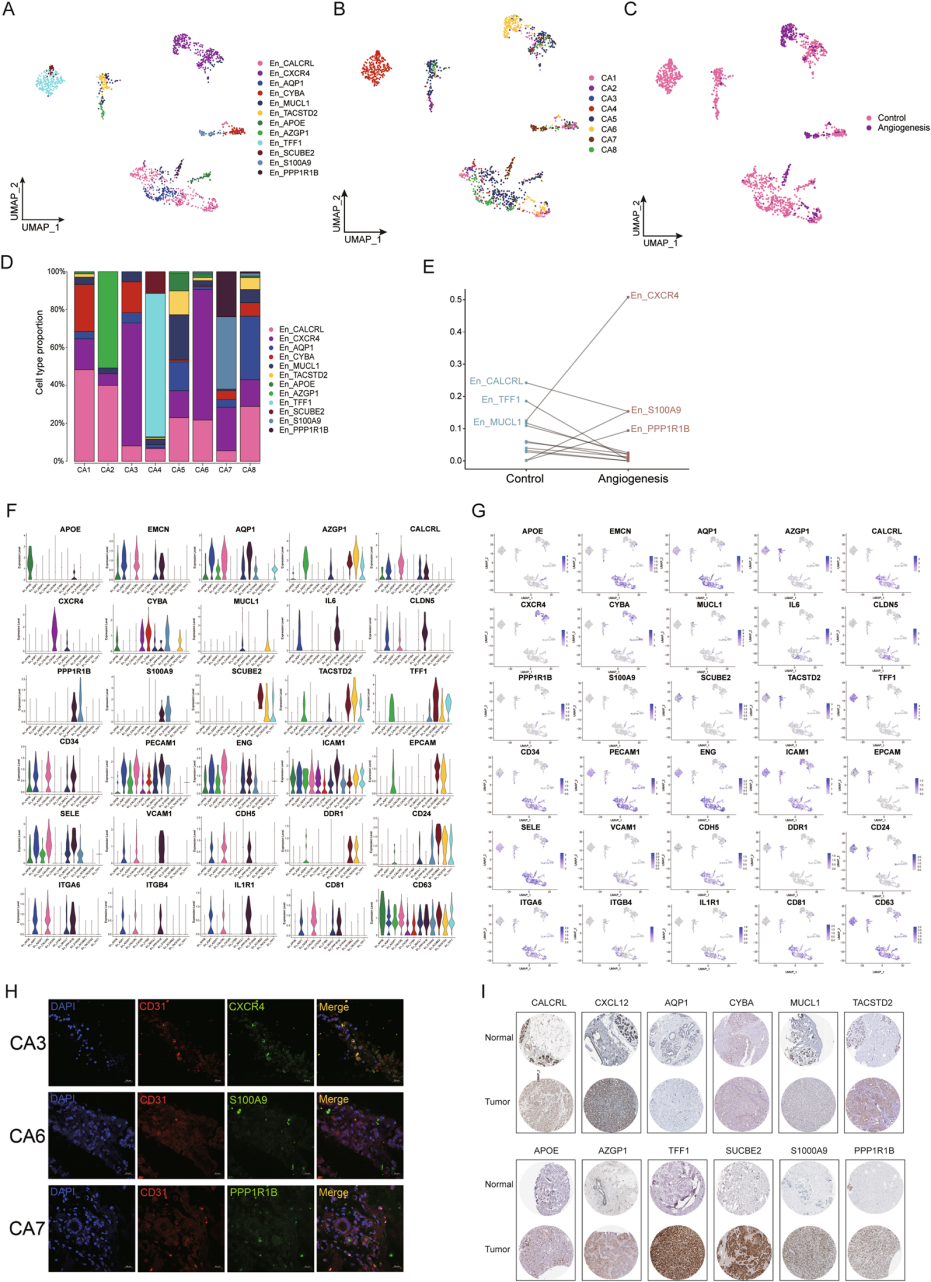
Characterizing endothelial cell subsets within the tumor microenvironment of breast cancer. **A** Single-cell map showing endothelial cell subsets. **B** UMAP plots of 1425 endothelial cells from eight patient samples. **C** UMAP showing single-cell profiles of endothelial cells in the angiogenic and non-angiogenic groups. **D** Endothelial cell subcluster composition for different patients. **E** Scatter plot showing differences in the distribution of endothelial cell subclusters between angiogenic negative and positive groups. The ecology of angiogenic and non-angiogenic breast cancer groups. **F**–**G** Violin plot and UMAP figure showing the expression of markers related to angiogenesis and exosomes in these endothelial cell subsets and individual distribution. **H** Immunofluorescence images of CXCR4, S100A9 and PPP1R1B expression, which are highly specific in the angiogenesis group and distribution of marker genes. Scale bar, 20 µm. **I** Representative IHC images of gene signatures

The heterogeneity of breast tumor-associated endothelial cell phenotypes across patients remains poorly inventoried at the single-cell level. TECs have significantly different biological characteristics compared with normal breast endothelial cells [37]. The heterogeneity of cancer cells is thought to be derived from clonal evolution. The origin of TECs and the effect of TECs heterogeneity still need further exploration. Gene profiles for each TEC subtype are correlated with distinctive functional programs and hold independent prognostic capability in clinical cohorts by association with metastatic disease. We identified 1425 endothelial cells in eight breast cancer tissues by single-cell sequencing and conducted a single-cell procedure using Seurat to identify cell clusters and marker genes on endothelial cell subclusters. We detected a total of twelve different cell types in the TECs cluster, these are En_CALCRL, En_CXCR4, En_AQP1, En_CYBA, En_MUCL1, En_TACSTD2, En_APOE, En_AZGP1, En_TFF1, En_SCUBE2, En_S100A9 and En_PPP1R1B (Fig. 5A). Endothelial cells from eight patients were visualized using UMAP (Fig. 5B). Endothelial cells formed twelve clusters that could be identified in all patient samples at varying proportions (Fig. 5C, D), implying intertumoral heterogeneity. In order to clarify the key endothelial cell subclusters that promote angiogenesis in breast cancer, we next performed differential expression analyses of each cell subcluster that varies highly in malignant endothelial cells with and without angiogenesis. These results showed that there are significant differences in expression levels between the two groups (Fig. 5E, Additional file 1: Figure S6).

In this study, we demonstrated these differences in endothelial cell types between the two groups, and we next examined several markers associated with blood vessels and extracellular vesicles transport. The UMAP reflected the distribution of these subclusters in the endothelial atlas (Fig. 5F, G). Tissue immunofluorescence staining was performed to investigate the distribution of these markers such as En_CXCR4, En_PPP1R1B, and En_S100A9 in breast cancer tissues and the relationship with the position distribution of blood vessels (Fig. 5H). We then obtained immunohistochemical images of these cluster-specific genes from endothelial cells from the HPA database. CALCRL, CXCR4, AQP1, CYBA, MUCL1, TACSTD2, APOE, AZGP1, TFF1, SCUBE2, S100A9, and PPP1R1B, were upregulated in breast tumor (Fig. 5I).

Above all, angiogenesis is a dynamic evolutionary process, the heterogeneity between ECs accounts for their roles in different stages of angiogenesis, some of them may play vital roles in this process as "switches". Moreover, small extracellular vesicles, act as intercellular communication agents between cells, its marker gene-CD63, was highly expressed in these endothelial cells. These results suggest that breast tumor cells and endothelial cells may be involved in complex interactions through small extracellular vesicles in the process of tumor angiogenesis.

### Small extracellular vesicles derived from breast cancer cells activated endothelial cells and promoted angiogenesis

**Fig. 6:**
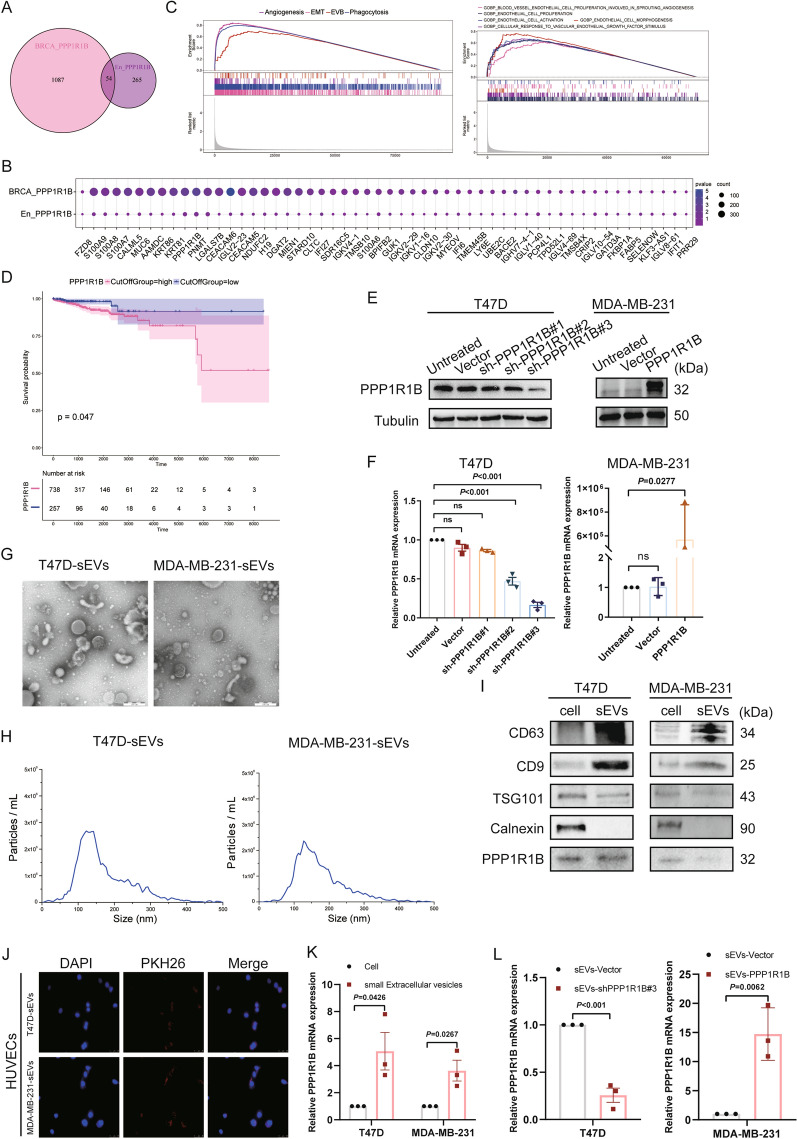
Single-cell atlas demonstrates shows association between specific endothelial and malignant cell subclusters in angiogenic breast cancer. **A** Venn diagram showing overlapping genes between BRCA_PPP1R1B and En_PPP1R1B. **B** Expression levels of common marker genes. **C** Gene set enrichment analysis (GSEA) showing the main pathways enriched in BRCA_PPP1R1B and En_PPP1R1B. **D** Effects of PPP1R1B expression on the overall survival of breast cancer patients. **E** Western blot analysis of PPP1R1B expression. **F** qRT-PCR analysis of PPP1R1B knockdown and overexpression efficiency. **G** Transmission electron microscope images of sEVs from T47D and MDA-MB-231 cells. Scale bar, 200 nm. **H** NTA showing the distribution of the size and concentration of isolated exosomes. **I** Western blot analysis of the expression levels of CD63, CD9, TSG101 (exosome marker genes) and calnexin (negative control) in sEVs and lysates from T47D and MDA-MB-231 cells. **J** Fluorescence microscope images showing the uptake of PKH26-labeled sEVs in HUVECs. Scale bar, 50 µm. **K** Relative expression of PPP1R1B in cells and sEVs. **L** Relative expression of PPP1R1B in T47D-sEVs and MDA-MB-231-sEVs after PPP1R1B knockdown and overexpression. Data were the means ± SD of three experiments. Statistical significance was determined by a two-tailed unpaired t-test (**K**, **L**)

PECAM1 was highly expressed in almost all endothelial subclusters, and all of these twelve clusters have high expression of CD63, including the endothelial cluster that was highly specifically expressed in the angiogenesis group—En_PPP1R1B [38, 39]. In addition, BRCA_PPP1R1B was also found in a high proportion of angiogenic breast cancer. Venn diagram analysis was performed to disclose 54 common genes for the interaction of BRCA_PPP1R1B and En_PPP1R1B (Fig. 6A). The expression levels of common genes were shown in the bubble map (Fig. 6B). BRCA_PPP1R1B displayed higher PPP1R1B mRNA expression levels when compared to En_PPP1R1B. GSEA analysis confirmed that BRCA_PPP1R1B is involved in signaling pathways such as angiogenesis, epithelial-mesenchymal transition (EMT), extracellular vesicle body, and phagocytosis. En_PPP1R1B is mainly involved in pathways related to the process of angiogenesis (Fig. 6C). This suggests that endothelial cell subclusters may have materials exchange with tumor cells and influence tumor angiogenesis through extracellular vesicle transport. The overall survival (OS) of the high-risk group was significantly shorter than that of the low-risk group (Fig. 6D). Then, T47D and MDA-MB-231 cells were used as “knockdown” and “overexpression” models of PPP1R1B. The transfection efficiency of PPP1R1B was verified by western blot and qRT-PCR (Fig. 6E, F, Additional file 1: Figure S7). A variety of evidences demonstrated that small extracellular vesicles could mediate intercellular communication among different compositions of the tumor microenvironment through transferring proteins, lipids, and nucleic acids [24]. In this study, we explored intercellular communication between breast cancer malignant cells and endothelial cells mediated by sEVs. We first isolated sEVs from the supernatants of T47D and MDA-MB-231 cells by differential centrifugation. Transmission electron microscopy and nanoparticle tracking analysis showed that T47D-sEVs and MDA-MB-231-sEVs were around with a diameter of about 40–160 nm (Fig. 6G, H). Western blot analysis showed that exosomes marker genes (CD63, CD9, TSG101) were detected in sEVs. And we found that PPP1R1B was presented in sEVs (Fig. 6I). To observe whether endothelial cells can take up tumor cell-derived sEVs labeled with PKH26, and the immunofluorescence results showed that endothelial cells could internalize sEVs successfully (Fig. 6J). This further suggests that endothelial cell subclusters may have materials exchanged with tumor cells and influence tumor angiogenesis through small extracellular vesicles transport. Here, we explored the expression and function of PPP1R1B in tumor cell-derived sEVs. We found that PPP1R1B was significantly elevated in sEVs (Fig. 6K). At the same time, the expression of PPP1R1B in sEVs decreased after PPP1R1B knockdown in cells (Fig. 6L).

**Fig. 7:**
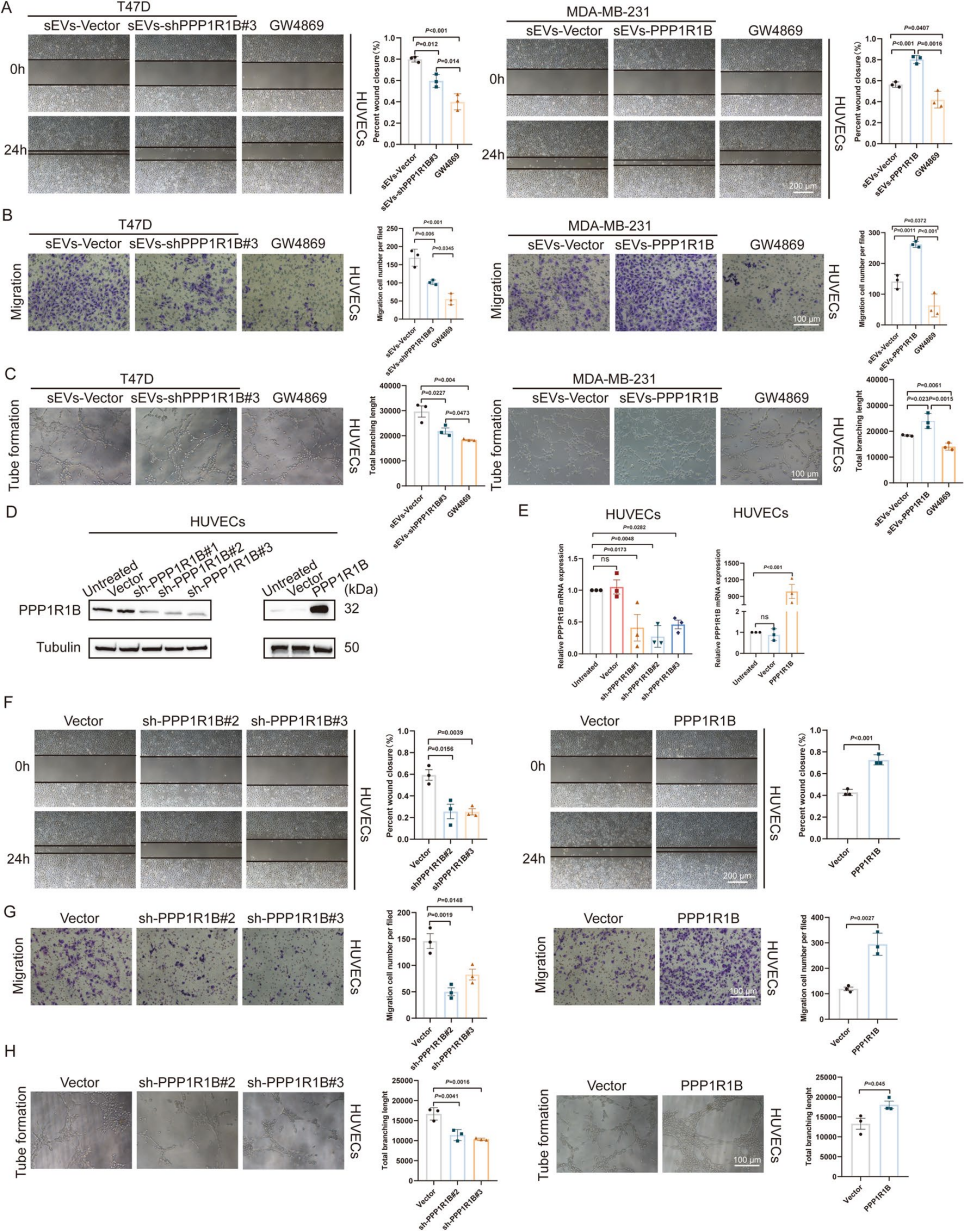
PPP1R1B promotes HUVECs angiogenesis. **A**, **B** The role of T47D and MDA-MB-231 cells derived sEVs cells on the migratory capacity of HUVECs. Representative micrographs of the wound healing and transwell assays. Scale bars, 200 µm and 100 µm. **C** Tube formation of HUVECs co-cultured with the sEVs-Vector, PPP1R1B-knockdown/overexpressed sEVs and GW4869. Scale bar, 100 µm. **D** Western blot analysis of PPP1R1B in HUVECs after transfection. **E** The expression of PPP1R1B was analyzed by qRT-PCR. **F**, **G** Wound healing and transwell assays were used to evaluat the migratory capacity of HUVECs after knockdown and overexpression of PPP1R1B. Scale bar, 200 µm and 100 µm. **H** Tube formation was performed to measure the angiogenic function of PPP1R1B in HUVECs. Scale bar, 100 µm. Data were the means ± SD of three experiments. The significant difference was evaluated with one-way ANOVA followed by the Bonferroni post hoc (**A**–**C**). Statistical significance was determined by a two-tailed unpaired t-test (**F**–**H**)

Breast cancer cells derived sEVs increased the migration of HUVECs and the migration ability can be inhibited by GW4869 (Fig. 7A, B). Tube formation assays were performed to detect whether PPP1R1B-derived sEVs affect angiogenesis. Compared with sEVs-shPPP1R1B, sEVs-Vector significantly increased the branching length of HUVECs. Treatment with sEVs-PPP1R1B induced the tube formation of HUVECs, and GW4869 inhibited the tube formation ability of HUVECs (Fig. 7C). To further determine if the PPP1R1B level regulated angiogenesis, we used HUVECs to construct PPP1R1B knockdown and overexpression models (Fig. 7D, E). Wound healing assay and transwell assay showed that PPP1R1B mediated migration of HUVECs was significantly increased (Fig. 7F, G). The tube formation ability of HUVECs was significantly increased when PPP1R1B was overexpressed (Fig. 7H).

Overall, we identified molecular differences between BRCA_PPP1R1B and En_PPP1R1B. PPP1R1B was downregulated in En_PPP1R1B compared to BRCA_PPP1R1B. PPP1R1B showed a negative prognostic role in breast cancer. The study discovered that sEVs could transfer PPP1R1B from malignant cells to endothelial cells.

## Discussion

The treatment of breast cancer, especially breast invasive ductal carcinoma, remains confusing until now [40, 41]. Despite HER2 as an ideal target for breast cancer treatment, 15–25% of patients will relapse, presenting a significant clinical challenge [42, 43]. Anti-angiogenic drugs are a class of targeted therapies which can inhibit tumor angiogenesis, normalized tumor blood vessels, and reprogram TME [6]. Tumor with high angiogenesis scores were significantly associated with metastatic recurrence, and were correlated with infiltration of immune cells [44]. Anti-angiogenesis targeting vascular ECs would be an effective approach for breast tumor therapy. Single-cell technology contributes to a better understanding of tumor cells in humans, it presents a high mutation burden and heterogeneity, and is highly adaptable. Previous studies on the single-cell sequencing of breast cancer analyzed heterogeneity for immune cells, epithelial cells and tumor cells [25, 45–47], and single-cell studies of TECs focused on cell types or changes in endothelial cell ratios following anti-angiogenic therapy [48]. However, the single-cell differences in the process of angiogenesis have not yet been reported. As we all know, angiogenesis is involved in dynamic changes, so our study provided a new perspective on investigating the heterogeneity of TECs and angiogenesis in breast cancer through single-cell technology.

In this study, we collected eight breast cancer tissues, including two cases of breast cancer patients with angiogenesis and six cases of breast cancer patients without angiogenesis. We first identified malignant cell subsets in the samples and compared the differences in gene expression between the non-angiogenic and angiogenic groups using the single-cell analysis method. Due to the influence of internal and external environmental factors, cancer cells of monoclonal origin often do not have identical karyotypes, and cells with different karyotypes have different survival and proliferation abilities [49]. Some cells are gradually eliminated during the selection pressures, while others develop a proliferative advantage and play a key role in tumor progression [50]. Therefore, in order to further investigate the dynamics of these cell subpopulations during angiogenesis, we conducted pseudotime and observed that several cell clusters such as BRCA_AGR3 and BRCA_SLC39A6 were highly expressed in the non-angiogenic group, but other cell clusters like BRCA_SRGN and BRCA_PPP1R1B were specifically expressed in the angiogenic group. These results suggest that SRGN and PPP1R1B may play vital roles in angiogenesis in breast cancer. In order to further figure out the process of gene expression changes during angiogenesis, the clonal evolutionary relationship was displayed by fish plot, the process was divided by three time points: develop naturally, vasculogenic mimicry, and angiogenesis. The expression levels of these malignant cell subsets showed significant differences at three distinct time points.

In the process of tumor angiogenesis, various internal and external factors promote angiogenesis through the induction of gene expression in endothelial cells, which can affect tumor growth and migration [51]. We identified first the malignant subpopulations associated with angiogenesis in tumor cell subpopulations. Furthermore, we divided endothelial cells into different subpopulations in order to further investigate the driving factors in the angiogenesis process. Nearly 1425 TECs were collected from breast tumor tissues, and twelve clusters were identified in our study. Several genes such as CXCR4, APQ1, TACSTD2, and S100A9 have been reported to participate in angiogenesis or are highly expressed in endothelial cells in cancer. However, others were first found to be involved in breast cancer angiogenesis. En_CXCR4, En_S100A9, and En_PPP1R1B were specifically highly expressed in the angiogenic group, suggesting that these marker genes may be new targets for anti-angiogenic therapy and forebode breast cancer prognosis in the future. The specific structure of blood vessels and the high expression of PPP1R1B in both tumor cells and endothelial cells may be responsible for the distant metastasis and progression of tumors [29, 32, 52–54]. Apart from that, we found these twelve tumor-associated endothelial cell subpopulations, in addition to high expression of the known vascular endothelial cell surface marker-CD31. Intriguingly, exosomal marker-CD63, and part of them express another exosomal marker-CD81 are also highly expressed in these endothelial cell subpopulations. A series of studies indicated that breast tumor cells and TECs exchanged reciprocal growth factors by sEVs. This unexpected discovery provides us with a completely new direction to study the relationship between sEVs and tumor angiogenesis, and the correlation between these genes and sEVs and angiogenesis needs to be further explored in future studies.

These results will allow us to elucidate the roles of TECs and sEVs in angiogenesis in breast cancer. We demonstrated that TME, especially one subset of them, angiogenesis, was the key factor in tumor growth and metastasis of breast cancer, and sEVs also played an important role in TME[55–57]. At present, anti-angiogenic therapy mainly relies on targeting VEGF/VEGFR or PDGF/PDGFR [6, 58]. However, these targets are just one of many factors in the process of angiogenesis in breast cancer, because the driving factors of angiogenesis are different among individuals, which may cause there to be no response for some patients to traditional anti-angiogenic therapy. TECs were detected in almost all breast cancer patients, and they were the major source of various validated protumor growth factors in the TME, targeting TECs marker genes may be an optimal choice for breast cancer treatment [45, 59]. Therefore, the important roles of these TECs in angiogenesis in breast cancer should be considered for the future studies.

Despite the significance of the findings in this study, there are still several limitations. First of all, although some evidence supports the hypothesis that the special tumor microenvironment leads to a unique TECs subpopulation, further studies are needed to characterize the evolutionary characteristics of this phenotype and to determine their involvement in the progress of breast cancer and related mechanisms in vivo and in vitro. Furthermore, due to the lack of spatial location, it is unclear whether differences in the location of the tumor would affect the result. The spatial distributions of tumor and endothelial cells in the progress of breast cancer angiogenesis deserve to be further investigated. Moreover, in the current study, we did not consider the effect of other cell clusters in the tumor microenvironment on tumor angiogenesis. More analyses are needed to improve the understanding of how TECs communicate with cell types and how new blood vessels are formed. In summary, we identified the expression profiles of subsets of cells in breast cancer and confirmed the characteristics of these TECs subsets. This cell atlas provides in-depth insights into breast cancer angiogenesis and its heterogeneity and is an essential resource for anti-angiogenic drug discovery in the future.

In addition, this study demonstrated that PPP1R1B was significantly associated with breast cancer prognosis. These results highlighted the importance of PPP1R1B as a valuable resource, as it was transmitted from malignant cell subclones to endothelial cells via sEVs. Once taken up by endothelial cells, PPP1R1B in turn promoted tumor angiogenesis and metastasis.

## Conclusions

By integrating scRNA-seq, we constructed a single-cell landscape from eight breast cancer tissues and found that PPP1R1B is specifically expressed in BRCA_PPP1R1B and En_PPP1R1B. And this study revealed that extracellular vesicles derived from breast malignant cells can potentially stimulate the formation of new blood vessels by activating endothelial cells through transferring PPP1R1B in the tumor microenvironment. This discovery provided a novel direction for anti-angiogenic therapy in human breast cancer, emphasizing the importance of exploring the role of extracellular vesicles in cancer progression and treatment.

### Supplementary Information

**Additional file 1: Figure S1.** UMAP view of cell subclusters in the malignant phenotype of angiogenesis. **Figure S2.** UMAP plots showing all cell types from control and angiogenesis breast cancer tissues. **Figure S3.** Cell composition of samples according to pathological types. **Figure S4.** The UMAP showing the distribution of each malignant cell subcluster in all malignant cells. **Figure S5.** Cancer cell composition of samples according to pathological types. **Figure S6.** Endothelial cell proportion of samples according to pathological types. **Figure S7.** Western blot showed the expression levels of PPP1R1B protein in various breast cancer cells.

**Additional file 2: Table S1.** Clinical information of patients.

## Data Availability

All scRNA-seq data will be provided upon request to the corresponding author Jing Hu (hujing@ems.hrbmu.edu.cn).

## Abbreviations

scRNA-seq: Single-cell RNA sequencing
PPP1R1B: Protein phosphatase 1 regulatory inhibitor subunit 1B
TECs: Tumor endothelial cells
TME: Tumor microenvironment
sEVs: Small extracellular vesicles
UMAP: Uniform manifold approximation and projection
CNVs: Copy number variations
SCENIC: Single cell regulatory network inference and clustering
GO: Gene ontology
KEGG: Kyoto encyclopedia of genes and genomes
HPA: The Human Protein Atlas
GSEA: Gene set enrichment analysis
IHC: Immunohistochemistry
OS: Overall survival
