# Supplementary material for: Single-cell RNA sequencing reveals small extracellular vesicles derived from malignant cells that contribute to angiogenesis in human breast cancers
Source: J Transl Med. 2023 Aug 25;21:570. doi: 10.1186/s12967-023-04438-3 (PMC10463655; doi:10.1186/s12967-023-04438-3)
Supplement: Supplementary file 1 — Additional file 1: Figure S1. UMAP view of cell subclusters in the malignant phenotype of angiogenesis. Figure S2. UMAP plots showing all cell types from control and angiogenesis breast cancer tissues. Figure S3. Cell composition of samples according to pathological types. Figure S4. The UMAP showing the distribution of each malignant cell subcluster in all malignant cells. Figure S5. Cancer cell composition of samples according to pathological types. Figure S6. Endothelial cell proportion of samples according to pathological types. Figure S7. Western blot showed the expression levels of PPP1R1B protein in various breast cancer cells. [file 12967_2023_4438_MOESM1_ESM.docx]

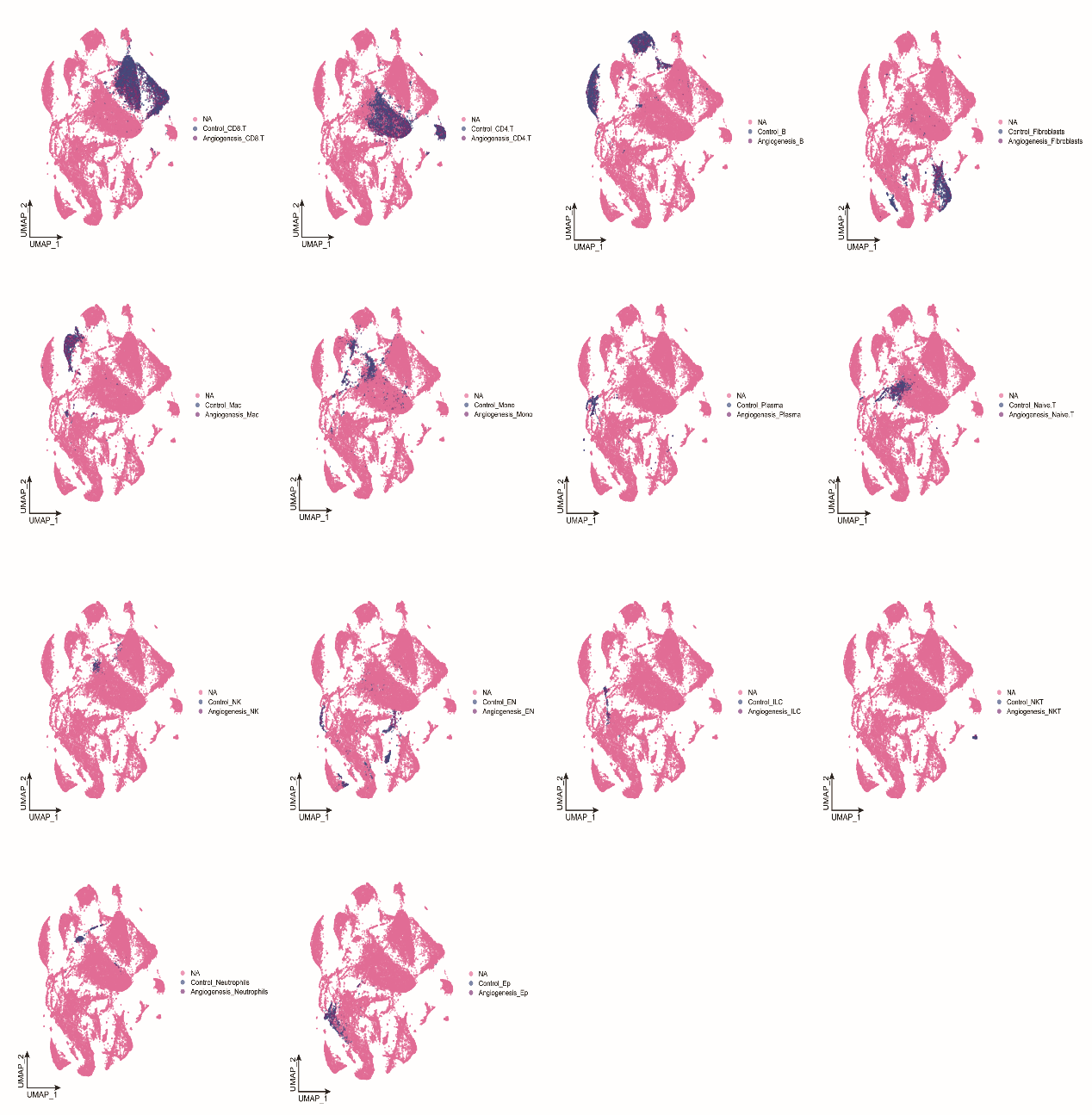


**Figure S1. UMAP view of cell subclusters in the malignant phenotype of angiogenesis****.**


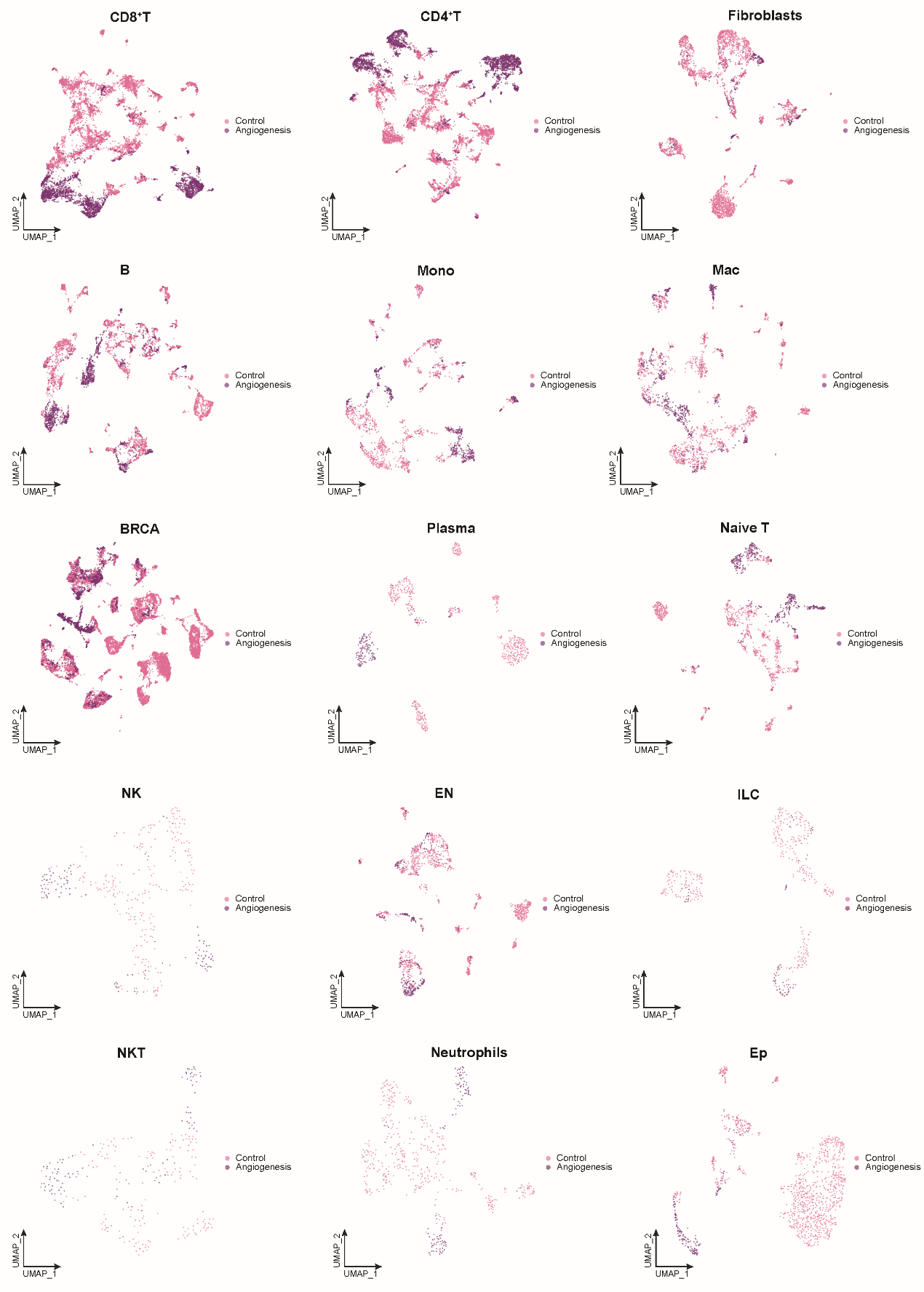


**Figure S2. UMAP plots showing all cell types from control and angiogenesis breast cancer tissues.**


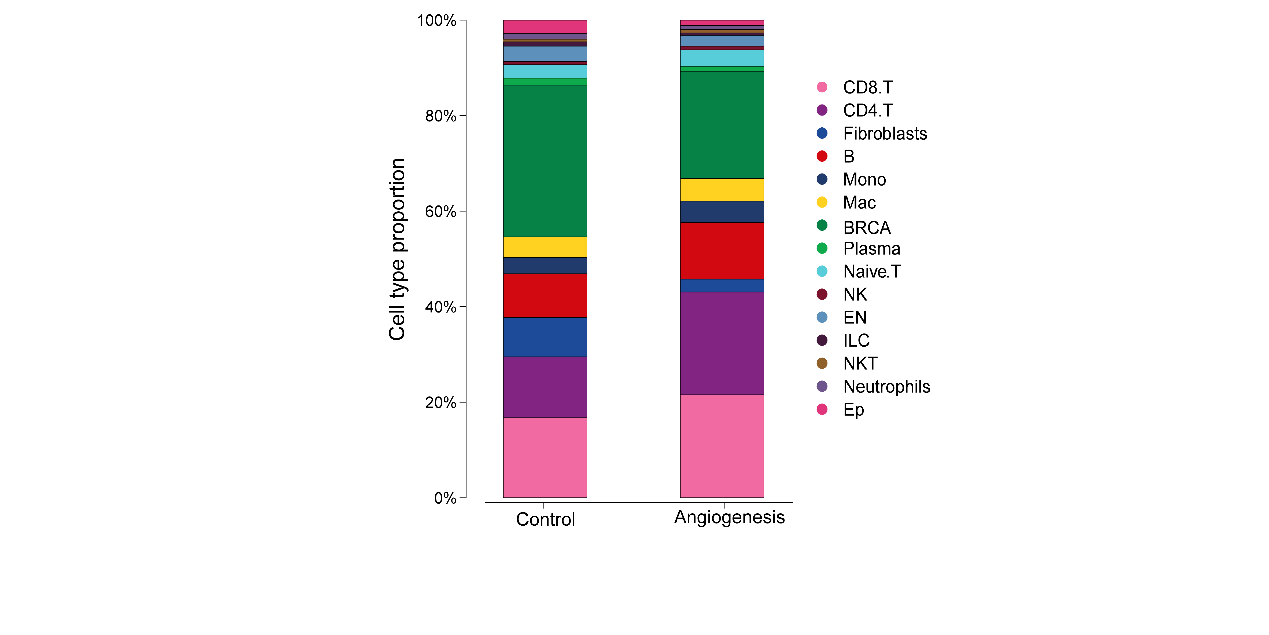


**Figure S3. Cell composition of samples according to pathological types.**

**
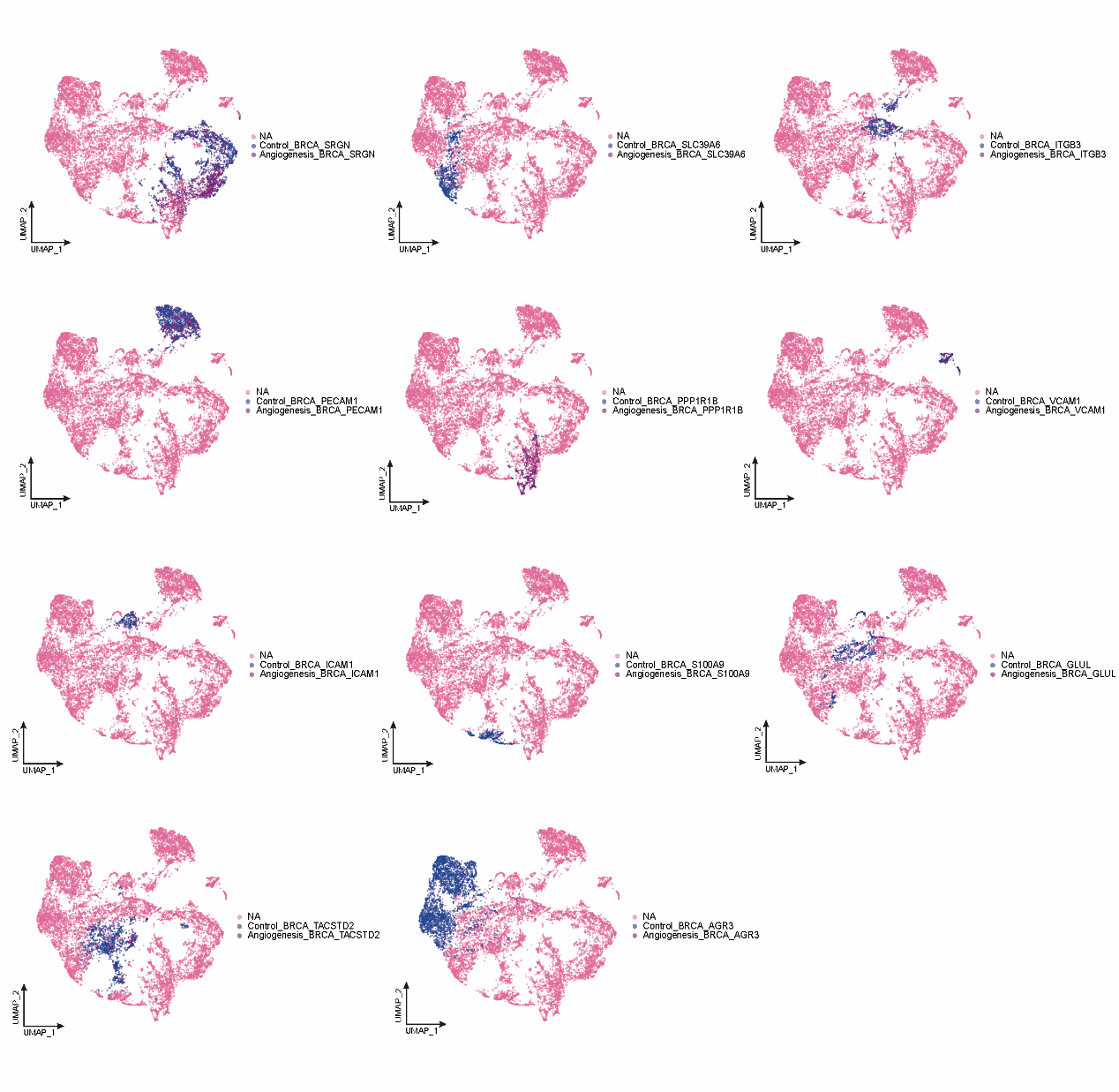
**

**Figure S4. The UMAP showing the distribution of each malignant cell subcluster in all malignant cells.**

**
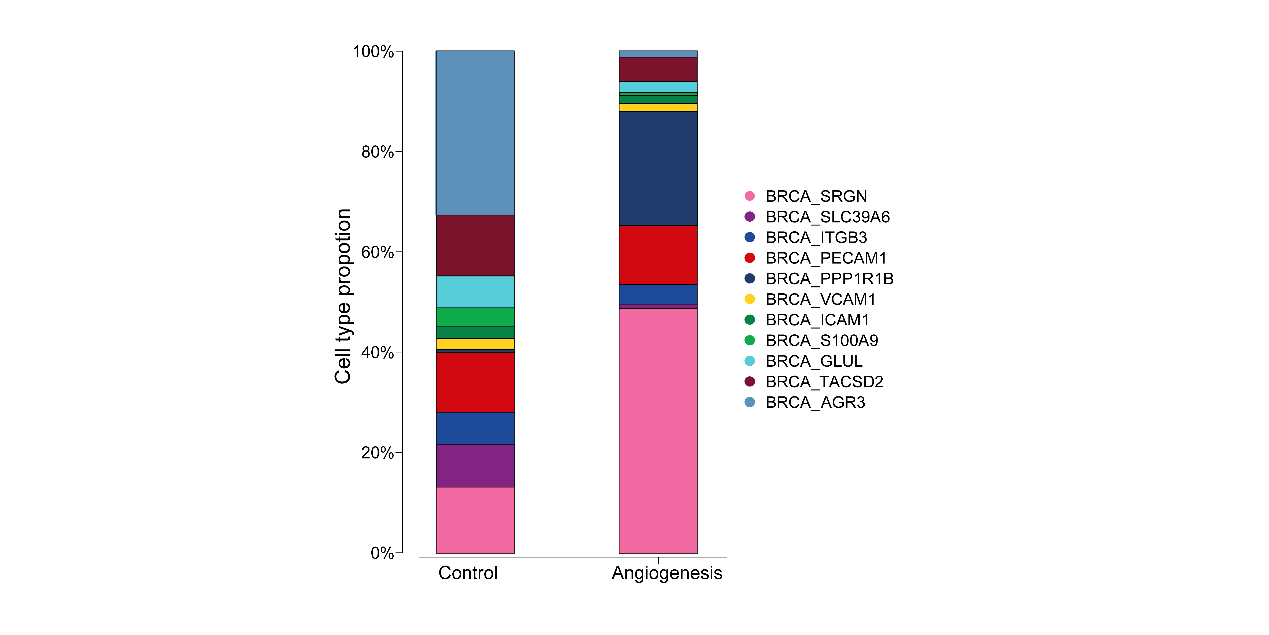
**

**Figure S5. Cancer cell composition of samples according to pathological types.**


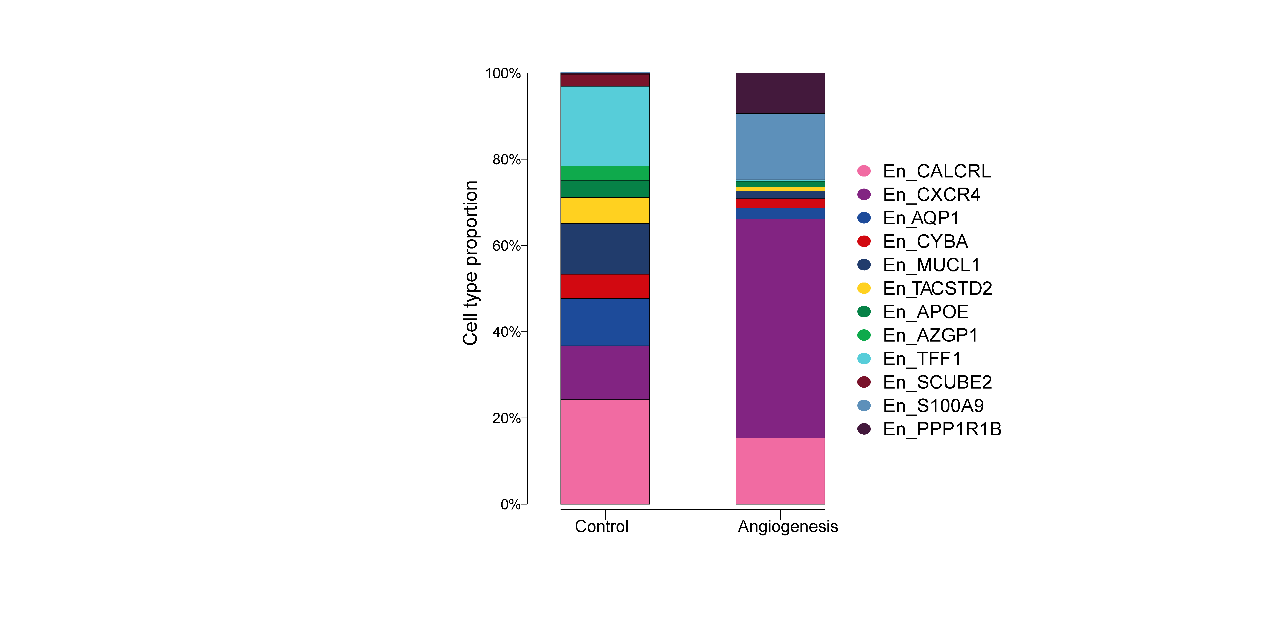


**Figure S6. Endothelial cell proportion of samples according to pathological types.**

**
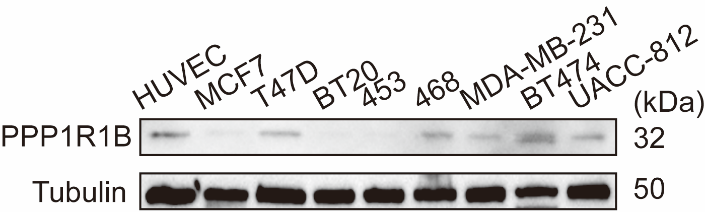
**

**Figure S7. Western blot showed the expression levels of PPP1R1B protein in various breast cancer cells.**
